# Notes and Comments

**Published:** 1922-07

**Authors:** 


THE
### HOSPITAL and HEALTH
REVIEW

### THE INCREASE OF CANCER.

ONCE more the increase of cancer has become a topic. Sir William Veno, whose recent munificent donation for cancer research will be remembered, has given in the " Times " some recent figures which show how great is this menace. In Great Britain the disease claims over 40,000 victims every year. In the United States the figure is over 80,000. In the quinquennial period 1912-1918 there was a total of 1,519,964 deaths, or an average rate of 71·6 per 100,000 of the population of the registration countries of the world. In Africa the number of deaths was 3,018, or a rate of 33·4. In America the figures were 251,438 and 65·7 respectively, in Asia 148,447 and 54·4, in Australasia 20,345 and 73·0, and in Europe 1,096,716 and 76·6.

Thus fully-developed countries, or those countries in which the population is largely white, have the largest death-rates. But if the figures are dissected, still more striking death-rates may be seen. Thus, though the rate for Africa is as low as 33·4, those of England and Wales and the Netherlands, two countries populated with highly civilised peoples, are as high as 94 and 93 respectively. Again, in Japan, although up to comparatively recent times the disease was almost unknown, it now claims as many victims as in England, and there seems to be little doubt that this state of affairs has arisen since the country has been Europeanised in customs and diet. Take the American statistics. The State of Massachusetts tops the list with a rate per 100,000 of 83·6, Rhode Island and New York coming next with rates of 82·7 and 80·4 respectively At the bottom of the list are Montana and Kentucky. with rates of 53·7 and 48·3. Wherever the negro population is highest the cancer death-rate is lowest Here, again, is evidence that white and highly civilised populations suffer more from the disease than semi-civilised and barbaric peoples.

Cancer is apparently a nemesis, following our departure from the simple life. It is due in some way to civilisation, and we must look to civilisation for the remedy. Is the disease connected with diet ? In his recent Olver-Sharpey lectures at the Royal College of Physicians on " Common Defects of Diet," Dr. E. Mellanby brought forward evidence of a dietetic origin. The Eskimos, before the introduction of modern diets, had no cancer, and the same was true of the Icelanders. Only with the introduction of these modern diets did cancer become common in Iceland, and it is now found actually among the Eskimo. But so far we know nothing of the *cause* of cancer in the precise sense of the term ; we only know something of the conditions under which it arises. How little we know is shown by the diverse causes suggested in the press by eminent men—tea-drinking, the cooking of food, inhalation of tar dust from the roads, and microzoa which grow in stored water. As to treatment, the only advance which has been made in recent years has been the use of the X-rays and radium. Though useful, these have only a restricted field. Early operation still is the mainstay.

### THE WATER SHORTAGE.

THE lay Press has been full recently of scare articles on the question of water shortage. So far as London is concerned, ill-informed criticisms have been printed, suggesting that special pipes should be laid to Wales in order to tap the supplies there, regardless of the capital expenditure involved, which would amount to millions.

Those centres, like Manchester, which draw their supplies from dammed lakes or natural gathering grounds, are on the whole in a favourable position. Districts like parts of Kent, which are dependent upon deep-seated sources of supply, may well find these supplies suddenly stopping, for no one really knows the true position underground of water reservoirs. A city like London, which draws 60 per cent. of its supply from the River Thames and 20 per cent. from the River Lea, depends partly on the flow of water in these rivers, which this year is better than in the corresponding period last year, and partly upon the scientific methods adopted by Sir Alexander Houston and his staff to render river water, polluted as it must be, safe for domestic consumption.

Assuming that no considerable quantity of rain falls for the next three months, if reasonable economy is exercised, no serious inconvenience need be expected in the Greater London area, but the consumption per head per day in that area is approximately 32 gallons, as compared with 22 gallons in Birmingham. The excess is largely the result of waste, and this will have to be stopped should the drought continue. Centres like Manchester and Liverpool, which have ample supplies in store, ought not to feel the pinch. But already 200 districts are suffering from an abnormal shortage, and in country villages especially the utmost care will have to be exercised, both as regards the quantity and the quality of the water available.

The absence of purifying rain is already beginning to have its effect on the health of the population. The absence of the flushing by steady rain of drains and ditches increases the danger of epidemics. From the technical point of view, the articles printed in the June number of this Review contain much valuable advice, and are very appropriate at the present crisis in districts where there is a serious shortage. Consumers should realise that careless waste of water at such a time as this is criminal. In country districts special care ought to be taken, as water easily becomes contaminated owing to cracks in the ground, and from other sources of pollution. Chlorination will certainly have to be adopted much more extensively. London water has been chlorinated since 1916 without any public complaint on the score of taste. There is no reason why similar precautions should not be taken in any part of this country.

### NEW HEALTH LEGISLATION.

THIS month it is probable that three measures affecting national health will be placed before Parliament, either by Sir Alfred Mond or under his auspices. Two months ago this Review foreshadowed the provisions of the Milk Bill which was introduced in the House of Lords by Lord Onslow, Parliamentary Secretary of the Ministry of Health. Its main purpose is to repeal the 1914 Milk Act, for which Sir Herbert Samuel was responsible—not, as the "Daily Mail" has stated, Dr. Addison, who was not at that time a member of the Cabinet at all. As well as repealing former legislation, the Milk Bill continues the experiment of grading milk, and increases the penalties for selling milk from tuberculous cows. Registration of dairymen will also be revocable.

So far as it is possible to ascertain, the Milk Bill will not meet with a happy reception. The small group of enthusiasts who favour grading as a panacea for all milk impurities will be pleased, but those who consider that much more drastic measures ought to be taken, will be disappointed. It is possible that small retailers will object to the proposal to make it possible to revoke the licences of dairymen, and that Sir Alfred Mond may be compelled to abandon this clause. He has made several attempts to obtain agreement on his Bill, and the result is the present compromise.

Another measure of considerable importance to the health of those who live in industrial centres is a Bill on the lines recommended by Lord Newton's Committee on Smoke Abatement. This will give the Minister of Health power to fix standards to govern the emission of "black" smoke from manufacturing chimneys. The onus of proof will lie on the manufacturer in any case in which the emission of smoke, grit or other noxious matter exceeds the standard so fixed. It is believed that such a measure, if properly administered by local authorities, will lead to substantial improvement. It may be introduced by Lord Newton in the House of Lords with favourable consideration by the Government, or else as a Government measure.

It is also probable that a small Bill dealing with lunacy reform will be brought forward before the summer vacation. The object of this will be to enable early treatment to be given without certification. The questions involved in this Bill are discussed at length by Dr. H. J. Norman on page 285 of this issue.

### THE TUBERCULIN TEST IN CATTLE.

IT is said to be an English characteristic to prefer concrete to abstract evidence, and certainly we are inclined to be, more impressed by a single example than by a statistical compilation, be the figures never so imposing. So we turn with relief from the statistical evidence—remarkably lacking in unanimity—as to the part played by the bovine tubercle bacillus in provoking disease in man to the following case, which teaches something about human nature as well as about tuberculosis. In Illinois,

U.S.A., one Robert Goben had his entire herd of cattle tested by tuberculin. "Black Lady," a thoroughbred cow, gave a positive reaction. She was, therefore, branded and placed in quarantine until such time as she could be slaughtered. Her owner, however, believed that a mistake had been made; the cow was in good flesh, gave rich milk, and seemed to be thoroughly healthy. So he was reluctant to have her killed, and when he left his farm he gave "Black Lady" to a farm hand who had seven children. The milk was very rich, and all the parties to the transaction considered it good. A little later "Black Lady" gave birth to twins, one of which soon died. Soon afterwards one of the children, aged five, fell ill, complaining of pain in the back, where a swelling was found. The physician who examined her suspected tuberculosis and began to investigate possible sources of infection. The child had apparently never been near anyone with tuberculosis, but on delving into "Black Lady's" past the physician discovered the tuberculin record and secured her destruction. The post-mortem examination showed that the healthy-looking cow that gave such rich milk was riddled with tuberculosis; both lungs, all the important glands in the body, the liver and udder showed tuberculous lesions. The tuberculin test was applied to a cow that had been stabled with "Black Lady" and to a pig, and, the reactions being positive, they were slaughtered, as well as four domestic cats. All six animals had generalised tuberculosis, and the pig and the cats had been fed with "Black Lady's" milk. A careful examination was now made of all the children. The eldest, who rarely drank milk, and the baby who had never been given cow's milk, were in good health. But the five who had been drinking "Black Lady's" milk had enlarged glands which were diagnosed as tuberculous. The afternoon temperature of all five was persistently raised. The donor of "Black Lady" was fined $150 for violation of quarantine regulations. A boom in tuberculin testing of cattle occurred in the locality. Such placards as: "The Milk Served Here Is From Tuberculosis-Free Cows" began to appear in windows of restaurants. The moral is that, though it is bad manners to look a gift horse in the mouth, it is wise to submit a gift cow to the tuberculin test.

### IMPERIAL RESEARCH.

THE report of the Advisory Committee on Research into Diseases in Animals, which was appointed in November, 1920, by the Development Commission, has just been issued. This report should be read in conjunction with that of the Colonial Office "Committee on Research in the Colonies." Both insist that the problem of disease and of health, whether in man, animal or plant, is in reality one The acquisition of the greater part of our knowledge of human and of veterinary medicine, both curative and preventive, has resulted from the use of identical scientific methods. The Committee of the Colonial Office looks to the Universities as training grounds for future investigators; that of the Development Commission to veterinary institutions and laboratories. The only independent institution in the United Kingdom in which research into animal diseases is made is the Royal Veterinary College, London, and the total State subsidy received by five of such colleges for research purposes last year was only £3,696. In the provinces the state of research into animal diseases is at present lamentable.

The Committee recommends the gradual establishment of a cadre of research workers under an advisory organisation of scientific experts, and considers that on both scientific and political grounds it is desirable that no demarcation should be drawn between research work in the United Kingdom and in other parts of the Empire. It is hoped in some quarters that arrangements will be made to link up research workers in human and animal diseases by providing better facilities for research and post-graduate study in connection with the Institute of Imperial Hygiene in London.

### THE NATIONAL LEAGUE FOR HEALTH, MATERNITY, AND CHILD WELFARE.

THE National League for Health, Maternity and Child Welfare, of Carnegie House, 117 Piccadilly, W.1, has issued its report for 1921, with some account of the work of its ten kindred organisations. Its convalescent home at Shooters Hill for 14 nursing mothers, which also takes in babies under two years of age, admitted 150 patients last year for an average stay of one month. Here several infant welfare centres endow cots for varying periods. The Marjorie Lumley Toddlers' Home at Maidenhead gave a change of air to 68 small London children, eight at a time, the average stay being five weeks; while at the Lady Forster Guest-House at Sydenham (which, unfortunately, had to be closed for lack of funds after nine months) 238 nursing mothers, wives of unemployed ex-Service men, were given a restful holiday at a cost of about 19s. a week each. A gift of £100 for a Tired Mothers' Holiday Fund financed a badly-needed change for 37 housewives. The League also runs a Babies' Hotel for children under seven of the professional class whose parents, for certain specified reasons, are unable to look after them. Training as nursery nurses is provided for twelve educated women, who pay 50 guineas for a year's course. There was a small deficit on the lecture department, one of the most important educational activities of the League. Additional funds are urgently needed that the useful work of all the departments may continue.

### THE EDUCATION OF THE DEAF CHILD.

IN an address delivered before the Child Study Society and published in the "Lancet," Mr. Macleod Yearsley, consulting aural surgeon to the London County Council, calls attention to the common disastrous mistake of regarding the deaf child as mentally defective. A boy of four can listen to, understand, and profit by what is told him and ask questions about what he sees. But the deaf child of this age can do none of these things and must have a very hazy conception of his surroundings; his mind remains undeveloped, and at five will be little different from that of a child of two. But by the modern method of oral training he can be taught to speak and his mind developed. "Almost all deaf children," says a well-known authority, Dr. Kerr Love, in his introduction to Miss Martin's excellent pamphlet, "What the Mother of the Deaf Child Can Do," "are mentally sound, and this is especially true of deaf-born children." The deaf child is merely a normal child who has lost his most important educational sense. Whether he has been born without it or has lost it by disease after birth does not matter. The hearing child goes to school at five with his physiological education well under way, but in England the deaf child need not attend school until seven, thus adding two years to his already severe handicap. In Scotland deaf children must attend school at five; in England they are allowed to attend provided the parents have paid grants for them. In Glasgow they can attend as early as three. Mr. Yearsley holds that the deaf child's education should begin at three. But the child may be so situated that it cannot be sent to school, and then, as Miss Martin has shown, the mother can successfully undertake its education. The home life is specially adapted for early training. But the ideal method is for the mother and the teacher to tackle the child together. Miss Martin's pamphlet should be in the hands of everyone—mother, teacher, or surgeon—who has to deal with the deaf child.

### TEACHERS AND SICK CHILDREN.

During the last few weeks there have been several ill-informed attacks against the Ministry of Health and the Board of Education with regard to the refusal of the Ministry to approve the Sevenoaks Hospital for the reception of children of school age suffering from acute hip disease from the County Councils of London, Middlesex, Surrey and Kent, unless one paid teacher is approved by the Board of Education. The Sevenoaks Children's Hip Hospital is comparatively small, and recently there were only eight children suffering from tuberculous disease, sent by local authorities. The remainder were either paid for privately or by voluntary bodies or Boards of Guardians. Ignorant critics have in fact placed their accusations out of proportion.

The question has a far wider importance. It is obviously necessary that physically defective children whose health may be improved by treatment should have opportunties of education. In this special case there was no insistence on certificated teachers being appointed, but the Board of Education considered that there should at any rate be one paid teacher responsible for the instruction of those children for whom local authorities were paying. The Central Committee for the Care of Cripples, which works in conjunction with the Central Council for Infant and Child Welfare, has recently issued a valuable report which deals fully with this question. It has drafted a scheme to promote the complete provision of both treatment and education for physically defective children throughout the country. It estimates that there are 125,000 children requiring treatment, and that, excluding London, there are only 1665 beds for children in places giving both treatment and education, and 21 schools for the physically defective accommodating 1,610 children. Its proposals are necessarily dependent upon financial consideration, and the Committee at the present time is appealing not only for funds, but for voluntary workers to undertake the after-care of the children who have left hospital.

The report further contains interesting evidence proving the wisdom of Sir Alfred Mond and Mr. Fisher in insisting that satisfactory arrangements should be made for the education of children of school age who receive treatment at such institutions. Dr. Hough writes that he has compared the working of the Ethel Hedley Orthopædic Hospital at Calgarth Park, Windermere, as a hospital pure and simple and as a hospital school. "The difference as regards the easy working of the place, the discipline, the happiness and well-being of the children under school control is incomparable. We have no friction whatever between medical and educational staffs, the two authorities recognising the enormous advantages of acting in unison."

### FREE MEALS FOR SCHOOL CHILDREN.

The Board of Education recently issued a circular to local education authorities on the subject of the provision of meals for school children that is of importance both from the point of view of economy and of public health. At present local education authorities may assist school canteens in providing meals on days when the school meets and other days. During the last year the number of free meals thus provided has expanded beyond all precedent. The cost has risen steadily from £54,167 in 1908-9 to £257,896 in 1920-21, and last year suddenly rose to £1,056,852. This year the total expenditure is to be rationed to a limit of £300,000.

There are 50 or more boroughs in England, including Chatham, Deal, Dover, Gravesend, Ramsgate, Margate, Maidstone and Winchester, which do not spend a farthing on school meals, whereas other boroughs appear to be pauperising the community by the supply of free food. There are cases where, by reason of lack of food, children are unable to take advantage of the education provided for them. The intention of providing free meals was originally educational, and was not intended to take the place of poor law relief. There are cases where the provision of meals is a great convenience to parents and of assistance to the children's health, but in many of these the parents could easily pay for the meals so supplied, and part of the duties of the canteen committees is to recover such amounts from parents. The whole system needs drastic overhauling, and Mr. Fisher has acted somewhat tardily in trying to remedy its abuses.

### THE COUNTY NURSING MUDDLE.

SIR CAMERON GULL, chairman of the Health Committee of the Berkshire County Council, lately gave a stimulating address to the Oxfordshire Nursing Federation on the subject of "Co-operation in County Health Administration." He complained of the appalling amount of overlapping, and urged the need for co-operation between voluntary and State services. The large number of private nursing associations, he said, should co-operate with the county associations, and these with the Queen's Institute. If the public authorities failed to co-operate with the Ministry of Health, six of the seven different people would be visiting the same invalid. The county should be mapped into reasonable nursing areas, each of which should be placed under the charge of a competent and well-trained nurse, who would be able to do all the nursing if the area was not too large. The present system was inefficient, and much money was spent on travelling. It was odd that there were no public funds for the training of nurses as nurses. More than a midwife was needed in a country district, a fact to which, perhaps, the Ministry of Health would awaken some day. It was generally believed, we may add, that the principal value of the Ministry of Health would be to co-ordinate our existing health and hospital services; and though it has its hands full with many schemes, it is probable that more immediate progress can be made by co-ordinating existing resources and reducing anarchy to order than by new developments grafted on to a system at present confused.

### HOME NURSING FOR INSURED PERSONS.

IT is only a few months since certain Approved Societies, by arrangement with the Queen Victoria's Jubilee Institute of Nurses, provided home nursing for their members. There was need for this benefit, which has advantages not only for the insured person, whose recovery is expedited by trained care, but to the society whose funds are relieved by the shortening of his illness. Last autumn nine societies, including the Post Office Employees' Approved Society, the Stock Exchange Clerks' Health Insurance Society, and the National Society of Operative Printers and Assistants, entered into an agreement, says the "College of Nursing Bulletin," whereby they pay the Institute at the rate of 1s. 4d. a visit per member (or 5s. a week after 30 visits), the local nursing association keeping a record of the number of visits paid. On Jan. 1, 1922, a second scheme came into operation, as the result of a further agreement with the Prudential and National Amalgamated Societies. Under this scheme the insured person is instructed by the district nurse to obtain a voucher from his society entitling him to nursing benefit. When seven or eight visits have been paid, and it seems likely that more than ten will be needed, she directs the patient to apply for a further voucher. These vouchers, signed by the district nurse and recording the number of visits paid, are sent with her monthly report to the Superintendent of the County Nursing Association, or the hon. secretary of the local association, as the case may be. At the end of the quarter the associations classify the vouchers according to the societies concerned, and forward them to the Q.V.J.I.N., which send them to the societies, accompanied by forms stating from what district they have been collected, and requesting payment. Accounts are settled quarterly by cheque, payment being made at the rate of 1s. per visit per member nursed. This sum "shall be taken to represent 75 per cent. of the cost of providing the nursing, it being open to the nursing association to collect not more than 4d. a visit from the member if they think fit (1s. 4d. being taken as representing the cost of a visit). A society may, however, make special arrangements with the appropriate association in regard to the nursing of any particular member or members within the area of the association." These agreements do not apply to midwifery or maternity nursing of insured persons, unless the incapacity continues after four weeks. A joint committee, representing Approved Societies and Nursing Associations, will deal with all details of working the scheme.

At the annual meeting of the Hearts of Oak Benefit Society on June 8, it was stated that, in return for the contribution of an agreed amount by the society, its State-insured members would have the services of nurses connected with Queen Victoria's Jubilee Institute for Nurses.

### THE DANGER OF COPPER POISONING.

DR. W. W. CHAMBERLAIN, Medical Officer of Health for Rawdon, in his quarterly report, draws attention to a menace to health. Several complaints have reached him that water, after passing through copper cylinders in the hot-water service, has been contaminated by copper, as shown in the green discoloration of sponges, brushes, &c. Samples of the water have been sent to the County Medical Officer, and the matter is still under investigation. He has frequently warned housekeepers of the risks run with a moorland water supply and lead service pipes unless care is taken to allow water to run freely through the pipes before drawing off for either drinking or cooking purposes, but this is the first complaint he has had of copper being in solution.

### THE PUBLIC HEALTH SERVICES.

WE would draw the attention of our readers to the series of interviews with local authorities which we begin to publish in this issue. The first article deals with public health services in Liverpool, a city that has set an example to other local authorities in various directions, notably in the way it has dealt with the clearance of slum areas. These interviews are written by a contributor whose experience and knowledge of the subject are exceptional. In the past not enough credit has been given to public health services for their work. It is hoped that this series will be illuminating to all students of health problems.

### A SELF-SUPPORTING HOSPITAL.

THE difficult position of the large proportion of the middle class who, on the one hand, are not fit subjects for charity, and, on the other, cannot pay the charges of the nursing home and the fees of the operating surgeon and other specialists when overtaken by the calamity which requires these, is only too well known. It has been referred to in the present controversy on the future of the voluntary hospitals, and again in the prolonged discussion in the Press on the corresponding difficulty produced in "Harley Street" because a large part of the old clientèle can no longer afford the fees in these times of financial stringency. The manner in which the difficulty has been met by the foundation of St. Chad's Hospital, Birmingham, an institution which unfortunately is quite unique in this country, is of great interest. In the *Times* Mr. D. J. Richards, the House Governor, has given the following description:—

This institution for paying patients (100 beds available) was founded in 1914 for the patient who cannot afford the heavy expense of a private operation and is not of the class for whom the voluntary hospital is intended. The institution is managed on strictly business lines to eliminate any form of charity. The medical staff consists of some 20 consultants, each of whom is a member of the staff of a Birmingham hospital (general and special).

The fees are arranged on an inclusive basis (within the means of the patient) to cover all necessary treatment recommended—medical, surgical, X-ray, bacteriological. Each member of the staff possesses the right to call upon any other member for assistance in the treatment without additional charge to the patient, the whole cost to the patient often being less than the amount which would be necessary to cover nursing charges alone for such a case in a private nursing home. The hospital, being built on modern lines (containing 60 small private wards, four large wards with accommodation for six patients each, and two large open-air balconies facing due south), offers to the consultant, with its two complete operating theatres, dispensary, pathological laboratory, and X-ray rooms, similar facilities to those obtainable at the best-equipped voluntary hospital. The patient is constantly under the observation of a resident medical officer and nursed by an experienced matron and fully-qualified nursing staff.

Seven years' experience of the working of St. Chad's, during which time nearly 7,000 in-patients, drawn from a particularly wide area, have availed themselves of the advantages offered, has led Mr. Richards to claim that the scheme: (1) meets the needs of the patient; (2) helps the voluntary hospital in relieving the hospital of this class of patient, and thereby reserving available beds for those for whom the voluntary hospital is primarily intended; (3) is an advantage to the consultant; (4) is an advantage to the practitioner, who can be invited to co-operate. Surely it is possible, says Mr. Richards, for a scheme to be formulated on these lines in the metropolis. We would add: "and in other centres."

### A HOSPITAL BEQUEST DISPUTED.

ANOTHER case of disputed bequest has come before the Scotch courts with reference to the residue of the estate, amounting to some £7,000, of the late Miss Catherine M. Adamson, of Portobello. The claimants were the trustees of the Royal Victoria Hospital Tuberculosis Trust, and the Lord Provost, Magistrates, and Council of Edinburgh as trustees of the Victoria Hospital for Consumption and Diseases of the Chest, Edinburgh. When the will was made, in 1898, the Victoria Hospital at Craigleith was owned and managed by a body now represented by the Tuberculosis Trust. But under the Edinburgh Corporation Order Confirmation Act of 1916 the hospital was transferred to the Corporation. In 1919 the Trust acquired a hospital at Liberton for tuberculous patients. Lord Ashmore decided that the bequest was destined for the work carried on by the Corporation in the Victoria Hospital. The grounds of his decision were, briefly, that the testatrix wished her bequest to be identified with the hospital at Craigleith, which was vested in the Corporation; and as he read the statutory agreement, when the Victoria Hospital was transferred to the Corporation the bequest was of the kind not carried to the Tuberculosis Trust under the general vesting clause, but was covered by the exception in favour of the Corporation. Unlike the case which we noticed recently in these columns, the dispute did not primarily arise from any want of care in the phrasing of the will, since at the time when it was made there was no reasonable cause to question the name of the beneficiary. It was not till 18 years later that the institution was transferred to another body, and not till 21 years later that the representatives of the original owners and managers acquired a hospital elsewhere. In these circumstances the testatrix may be acquitted of causing an unnecessary lawsuit, though possibly—it is a question of dates—a codicil might have been drawn to redefine her intention in the altered circumstances.

### INSTITUTIONAL CARE OF THE AGED.

A PAPER which should be carefully read by all who are interested in the institutional care of the aged has lately been published by Dr. R. J. C. Thompson and Dr. R. T. Todd in the "Lancet" (April 29). The writers, who have been in medical charge of 500 veterans at the Royal Hospital, Chelsea, are convinced that much invalidism among the aged is unnecessary and is due to that loss of self-reliance and self-respect which indiscriminate coddling entails. Many old people are easily persuaded to take a morbid interest in being helpless and soon learn to enjoy being the coddled centres of attraction; the result is the self-centred abstraction so common in the aged. As long as a man responds to the "elementary stimuli"—self-reliance, self-preservation and self-respect—he does not *suffer* from old age. Mere physical deterioration is a very different matter from physical deterioration plus lowered mentality, and there can be little doubt that hospital surroundings tend to induce moral, as well as physical, deterioration in the aged. "Do anything for an old man which he can do for himself, and he will very soon persuade himself that he can no longer do it." As far as possible the aged should be kept out of bed, which encourages dirty habits and favours hypostatic pneumonia, particularly when chronic bronchitis, emphysema and the like already exist. A day in bed, with hot-water bottles and easily digested food, often tides over an impending crisis, reinforcing the threatened reserve of vital energy when the aged feel tired or "off their food," but this is a measure very different from an indefinite sojourn in bed. Cheery optimism in all concerned with the aged is invaluable, and the writers refer to the case of a Mutiny veteran, aged 89, who stated on admission to hospital, "This time I am booked for the Other World." He was assured that "the return half of his ticket" was sticking out of his pocket, and he made an excellent recovery from the broncho-pneumonia from which he was suffering. The unofficial tonic of cheery chaff is wonderfully effective, and the misapplication of so-called "sympathy" does nothing but demoralise old age. A visitor's moist eye and mournful shake of the head have never helped either the patient or those responsible for his welfare. In the Infirmary at Chelsea permanent hospital patients are allowed to smoke in bed, and their desires for small luxuries are humoured as far as possible. Incontinence, with no organic basis, is cured with astonishing frequency by withholding for a day one of the minor privileges, pipe or beer. The man associates the want of his pipe with the necessity for not soiling himself. A craving for coddling is not a universal feature of the aged, and one of the writers' patients was a Crimean veteran of 86, whose attitude towards the attentions of the other sex was expressed with more directness than courtliness in his ejaculation, "I wish these blasted women would leave me alone."

### MAKING HISTORY AT A MENTAL HOSPITAL.

THE stagnation and lack of opportunities imposed by the mental hospital service upon assistant medical officers were raising a protest even before the war. The slender chances of promotion, the discouragement of study, the virtually enforced celibacy of these unhappy men were keenly felt, and an organisation was even started to improve them. We trust that a sign of change is to be inferred from a modest sentence in the report of the visiting committee of the County Mental Hospital at Parkside, Macclesfield, which records that one of the assistant medical officers has received "permission to marry and to occupy the caretaker's house (unfurnished) at the Isolation Hospital as part of his emoluments." The report significantly adds: "This is the first occasion on which a junior assistant medical officer has been permitted to marry whilst in the committee's service." Thus has history been made at Macclesfield. To increase the chances of promotion, to encourage the young assistant medical officer to take an interest in the scientific, as opposed to the routine, side of his work may be more difficult than to allow him the amenities of matrimony. But the will must come before the way; and it is natural to make the simplest reform first. We congratulate the committee on this beginning, and the assistant medical officers on this recognition of their needs. When asylums live fully up to their new style of "mental hospital," they will cease to be the grave of promising young medical men.

### THE SPIRIT OF THE STAFF.

MR. JOSEPH MELLOR, Chairman of the House Committee of the Queen's Hospital for children, lately remarked on the new spirit which, since the war, seemed to dominate hospital staffs. The hospitals, he said, depended upon the goodwill of their employees; and to-day, whether he (or she) was employed by a hospital or not, an employee demanded a fair day's pay for a fair day's work. It was therefore impossible to reduce expenditure. What a light this throws upon the state of hospitals before the war! The best men in the hospital world always maintained that a voluntary hospital should set the highest standard in everything, including the wages paid and the consideration shown to its employees. Yet nurses, for example, were underpaid and overworked; nor is it desirable that such a state of affairs should be revived. But if the hospital spirit is to permeate the conditions under which hospital staffs work, it must also permeate the mood in which that work is done. A hospital is a place where the letter has often to be sacrificed to the spirit; accidents do not begin to arrive at nine in the morning, and cease to arrive at half-past six at night. Consequently the mechanical interpretation of union rules is impossible. But we should be sorry to believe that, as Mr. Mellor's words half suggest, any chairman thought hospital staffs should be underpaid because they work in a hospital. Fair pay for fair work should be an axiom everywhere, and the last place where it should be infringed is within a hospital's walls.

### HOSPITAL PORTRAIT GALLERIES.

THE board-room of the East Suffolk and Ipswich Hospital is what all board-rooms ought to be, a picture gallery. The walls are hung with portraits of the chief workers for the hospital, in which the Cobbold family is represented in many frames. To those already there has now been added the portrait of Mr. John D. Cobbold, who was Chairman from 1914-21, throughout the momentous changes that took place during the war. In that time, to summarise these, the beds rose from 140 to 300. In addition to his successful administration, Mr. Cobbold has been a very generous benefactor to the hospital; patients, nurses, children have all received benefit from his hands. From the foundation of the hospital in 1835 to the present time his grandfather, his uncle, his father and himself have all been active workers for it; and the Cobbold portraits, like the Cobbold Wing, enshrine a cherished family tradition.

